# Treatment With Voclosporin and Anifrolumab in a Patient With Lupus Nephritis and Refractory Discoid Lupus Erythematosus: A Case Report and Literature Review

**DOI:** 10.7759/cureus.55321

**Published:** 2024-03-01

**Authors:** Ralina Karagenova, Ziga Vodusek, Rebecca Krimins, Adam Krieger, Homa Timlin

**Affiliations:** 1 Rheumatology, University of Hawaii John A. Burns School of Medicine, Honolulu, USA; 2 Rheumatology, Johns Hopkins University School of Medicine, Baltimore, USA; 3 Radiology and Radiological Science, Johns Hopkins University School of Medicine, Baltimore, USA

**Keywords:** systemic lupus erythematosus, sle, anifrolumab, voclosporin, sle and lupus nephritis, lupus nephritis, discoid lupus erythematosus (dle)

## Abstract

Systemic lupus erythematosus (SLE) is a complex heterogeneous disease with multiple clinical manifestations. Recently, two medications, anifrolumab and voclosporin, have been approved for the treatment of adults with SLE and lupus nephritis (LN), respectively. We present the case of an elderly woman with LN and refractory discoid lupus erythematosus (DLE), who was treated successfully with a combination of voclosporin and anifrolumab without major infections.

## Introduction

Renal involvement adds significantly to systemic lupus erythematosus (SLE)-associated mortality and morbidity [1]. The most common type of chronic lupus rash is discoid lupus erythematosus (DLE), accounting for 73 to 85 percent [2,3]. DLE is characterized by well-defined inflammatory plaques that evolve into atrophic, disfiguring scars.

Severe DLE may be refractory to traditional immunosuppressants. Persistent use of glucocorticoids can lead to irreversible organ damage. Non-white patients with SLE tend to have more severe comorbidities than white patients [4].

Voclosporin is a calcineurin inhibitor that was approved for the treatment of lupus nephritis (LN) in 2021 [5]. Anifrolumab is a human monoclonal antibody against type 1 interferon receptor (IFNAR) that was recently approved for the treatment of moderate to severe SLE [6]. There exists a lack of evidence of combination treatment with voclosporin and anifrolumab. We report the first case of a patient with DLE and LN who was successfully treated with voclosporin and anifrolumab.

## Case presentation

**Figure 1:**
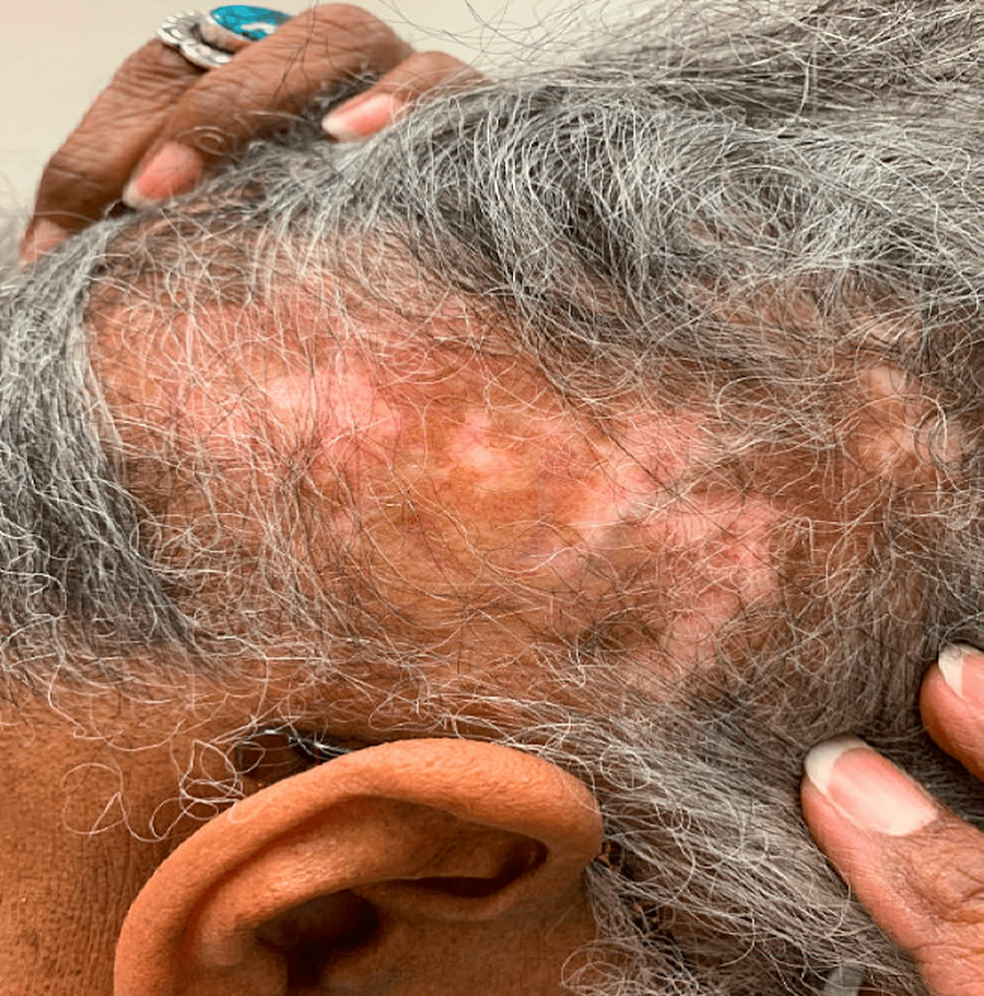
Active discoid lupus erythematosus

**Figure 2:**
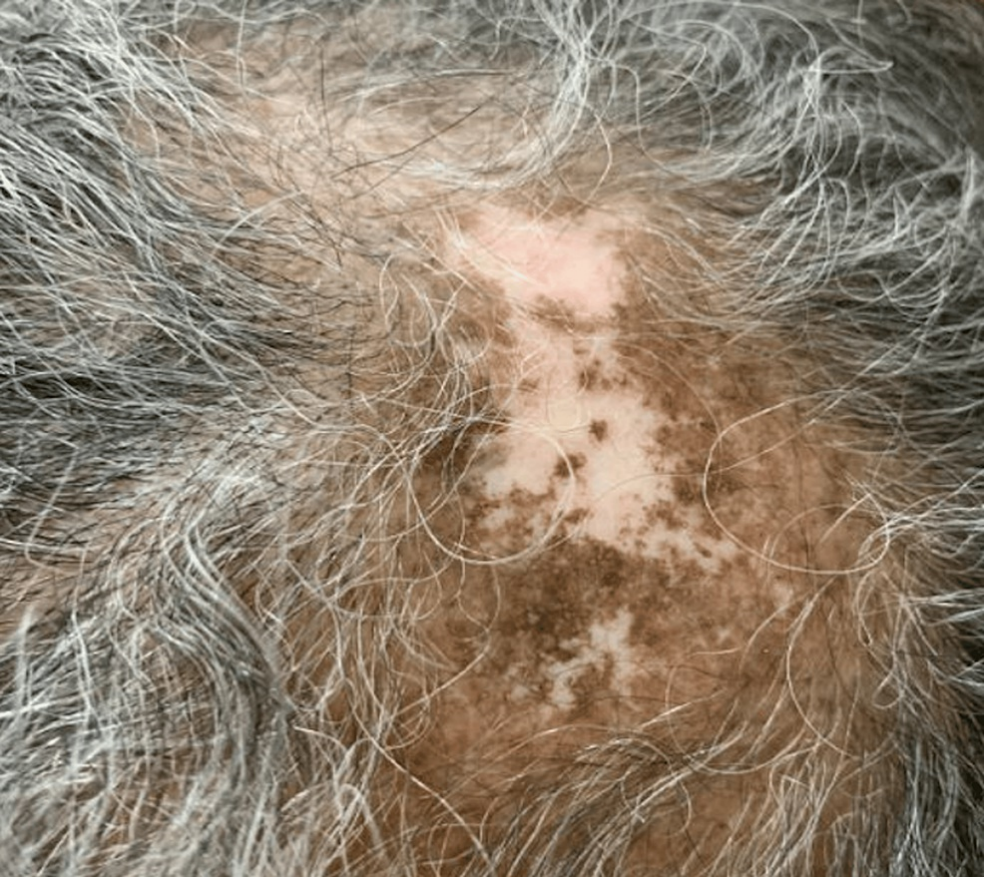
Healed discoid lupus erythematosus

A 69-year-old African American woman was diagnosed with SLE in her 30s characterized by positive antinuclear antibody (ANA), double-stranded DNA, Smith, arthritis, pleurisy, alopecia, mucosal ulcers, and DLE. Over the years, she had frequent flares of arthritis and DLE. She was subsequently treated with variable doses of glucocorticoids, hydroxychloroquine, mycophenolate mofetil, methotrexate, azathioprine, and baricitinib. Approximately two years ago, while on baricitinib, she developed proteinuria (peak 7g), and a kidney biopsy confirmed lupus nephritis class V. Within the first six months, after switching baricitinib to voclosporin (23.7 mg twice daily), her proteinuria normalized to 0.16 (normal 0.00-0.19 mg/g creatinine). However, her DLE remained active (Figure 1), and required frequent intralesional triamcinolone injections to the scalp every two to three months. At this time, she was started on monthly anifrolumab infusions. By the third month of infusions, the patient’s DLE had improved (Figure 2). The patient was able to taper off prednisone and no further triamcinolone injections were required. She had no further proteinuria and her arthritis remained under control. While on anifrolumab, the patient had one episode of a urinary tract infection, which resolved with a course of antibiotics.

## Discussion

The presence of lupus nephritis significantly reduces survival to approximately 88% at 10 years, with even lower survival in African Americans [7,8]. Furthermore, continued proteinuria with no remission is predictive of poor outcomes in most studies leading to high rates of chronic kidney disease and eventual progression to end-stage renal disease. DLE is also a debilitating condition that negatively impacts the quality of life [5]. 

Treatment options for LN and DLE are limited. Proteinuria can be reduced as rapidly as within two weeks as demonstrated in the voclosporin AURORA trial [6]. First-line DLE treatments include lifestyle changes, such as photoprotection and smoking cessation, in conjunction with topical glucocorticoids and topical calcineurin inhibitors. However, systemic treatment is often necessary. Two phase-3 studies (Treatment of Uncontrolled Lupus via the Interferon Pathway (TULIP)-1 and TULIP-2) [9] and a phase 2b study (MUSE) [10] offer considerable evidence for the efficacy and safety of anifrolumab for moderately to severely active SLE with observed benefits in cutaneous SLE.

Our patient had active DLE and failed multiple standard therapeutic interventions including topical, antimalarial therapy and disease-modifying drugs (DMARDs). Her proteinuria resolved on combination therapy with mycophenolate mofetil and voclosporin. However, she required frequent intralesional steroids. She showed remarkable improvement after three infusions of anifrolumab as an add-on therapy. Additionally, she managed to taper off steroids (oral and intralesional). Furthermore, she did not have any major infections or other adverse effects, the risk of which has been shown to increase with age.

## Conclusions

We present the first case of a patient with LN and DLE who was able to achieve remission of both conditions with voclosporin and anifrolumab. The patient has not experienced any major infections since the initiation of treatment. Furthermore, she was able to taper off prednisone, and intralesional triamcinolone injections were halted. There remains a gap in the literature on the safety and efficacy of anifrolumab in elderly patients with SLE. Future clinical trials are needed to investigate the safety of the combination of voclosporin and anifrolumab in elderly lupus patients and whether combination therapy can replace steroid use.
